# Assessment of induced allelopathy in crop-weed co-culture with rye-pigweed model

**DOI:** 10.1038/s41598-024-60663-w

**Published:** 2024-05-07

**Authors:** Waseem Mushtaq, Marie-Laure Fauconnier, Caroline de Clerck

**Affiliations:** 1 Laboratory of Chemistry of Natural Molecules, Gembloux Agro-Bio Tech, Liege University, Passage des déportés 2, 5030 Gembloux, Belgium; 2 AgricultureIsLife, Gembloux Agro-Bio Tech, Liege University, Passage des déportés 2, 5030 Gembloux, Belgium

**Keywords:** Secondary metabolism, Plant molecular biology

## Abstract

This study evaluates induced allelopathy in a rye-pigweed model driven by rye’s (*Secale cereale* L.) allelopathic potential as a cover crop and pigweed’s (*Amaranthus retroflexus* L.) notoriety as a weed. The response of rye towards pigweed’s presence in terms of benzoxazinoids (BXs) provides valuable insight into induced allelopathy for crop improvement. In the 2 week plant stage, pigweed experiences a significant reduction in growth in rye’s presence, implying allelopathic effects. Rye exhibits increased seedling length and BXs upsurge in response to pigweed presence. These trends persist in the 4 week plant stage, emphasizing robust allelopathic effects and the importance of different co-culture arrangements. Germination experiments show rye’s ability to germinate in the presence of pigweed, while pigweed exhibits reduced germination with rye. High-performance liquid chromatography with diode-array detection (HPLC-DAD) analysis identifies allelopathic compounds (BXs), 2,4-dihydroxy-1,4-benzoxazin-3-one (DIBOA) and 2,4-dihydroxy-7-methoxy-1,4-benzoxazin-3-one (DIMBOA) in rye. Rye significantly increases BX production in response to pigweed, age-dependently. Furthermore, pigweed plants are screened for possible BX uptake from the rhizosphere. Results suggest that allelopathy in rye-pigweed co-cultures is influenced by seed timing, and age-dependent dynamics of plants’ allelopathic compounds, providing a foundation for further investigations into chemical and ecological processes in crop-weed interactions.

## Introduction

We live in a world where agricultural productivity plays a pivotal role in ensuring food security and environmental sustainability. In this context, managing the interactions between crop plants and weeds takes center stage, and understanding the mechanisms governing these interactions is of paramount importance. Resource competition and chemical interference are two mechanisms of interaction among plants that can have negative effects on their performance and coexistence. Resource competition refers to the negative impact that one organism has on another by using up a shared resource, such as food or space^1^. This can lead to a decrease in the performance of the affected organism, as it has less access to the resources it needs to survive and grow. On the other hand, chemical interference refers to the negative effects that one organism has on another through the release of chemical compounds^1^. This can occur through allelopathy, where one species alters the abundance or distribution of another species through the release of chemicals (allelochemicals), allowing its population to increase and negatively affect other species^2^. While competition between plants for resources can trigger allelopathic responses, allelopathy itself is a broader phenomenon where plants release chemicals to influence other organisms, not just in competitive scenarios^2^. Allelopathy serves various ecological roles, including defence mechanisms, resource allocation, and ecosystem regulation. The allelochemicals released by a plant (donor) may interfere with physiological functions or impede seed germination, root growth, or other processes in nearby plants (recipient). Recently, allelopathy induced by the presence of weeds has been described in wheat, sorghum, rice and buckwheat^2,3^. Allelopathy can be induced by different means including competitor weeds’ root exudates^4^. Molecules from root exudates such as jasmonic acid, methyl jasmonate and (–)-loliolide have been shown to induce allelopathy in wheat paired with several common weeds^5^. Such induced allelopathy in crop plants can consume less energy by synthesizing herbicidal molecules only when the plant needs it.

Among allelochemicals, there’s a group known as benzoxazinoids (BXs), which are released by the roots of certain grass species^6^. BXs serve a dual purpose: they protect the plant from pests and diseases while also inhibiting the growth of neighbouring plants^7^. When released, BXs start as glucosides and then turn into aglycones, like the BXs 2,4-dihydroxy-(2H)-1,4-benzoxazin-3(4H)-one (DIBOA), 2,4-dihydroxy-7-methoxy-(2H)-1,4-benzoxazin-3(4H)-one (DIMBOA) and 6-methoxy-benzoxazolin-2-one (MBOA) which are more potent allelochemicals in crops like wheat, maize, and rye^8,9^. However, even though it is known that BXs can affect weed growth, information is lacking about their release from plant roots and whether neighbouring plants’ roots can take them up. These compounds, which exist in glycosylated or free form, exhibit significant diversity and are readily degradable both biotically and abiotically.

To increase knowledge in this field, we have decided to work on the plant pair rye: pigweed, using sandglass as a growing medium to mimic soil while avoiding the adsorption of BXs to soil particles. The crop we chose to focus on, rye (*Secale cereale* L*.*) is well-known for its allelopathic properties^10^. Rye produces allelochemicals notably through its root exudates (e.g. phenolics and benzoxazinoids)^9–12^. On the other side, pigweed (*Amaranthus retroflexus* L.) is a notorious weed that can outcompete crops, leading to yield losses^13–15^. Its ability to suppress the growth and lower the yield of plants of agronomic significance has been investigated and demonstrated in field tests, such as those involving sugar beet (*Beta vulgaris*)^16^, red kidney bean (*Phaseolus vulgaris*)^17^, and maize (*Zea mays*)^18^. Investigating the allelopathic interactions between these two species can offer valuable insights into strategies for weed management and crop yield improvement. In the initial stage of our study, we aimed to determine the conditions under which pigweed growth was affected most by the presence of rye or when rye imposes its maximum allelopathic potential in rye-pigweed co-culture at different growth stages. We also sought to find the co-culture patterns in which pigweed induces allelopathy in rye. We are curious to know if the sowing time of seeds may impact the allelopathic potential of rye by affecting the timing of the allelochemical release, which can suppress pigweed growth. Optimizing sowing time in a crop-weed model can enhance allelopathic interactions, leading to improved weed suppression and overall crop productivity. Moreover, we investigated changes in the concentration of BXs in the rye plant in response to co-cultivation with pigweed. Furthermore, we explored the root uptake of BXs by pigweed and their transport to the shoot. This multi-faceted approach allowed us to comprehensively explore the allelopathic dynamics within this co-culture system. This study establishes a foundation for further investigations into the chemical and ecological processes at play in crop-weed interactions, ultimately contributing to our understanding of these complex agricultural systems.

## Results

### Seedling length and dry biomass allocation of 2 week-old plants

**Figure 1:**
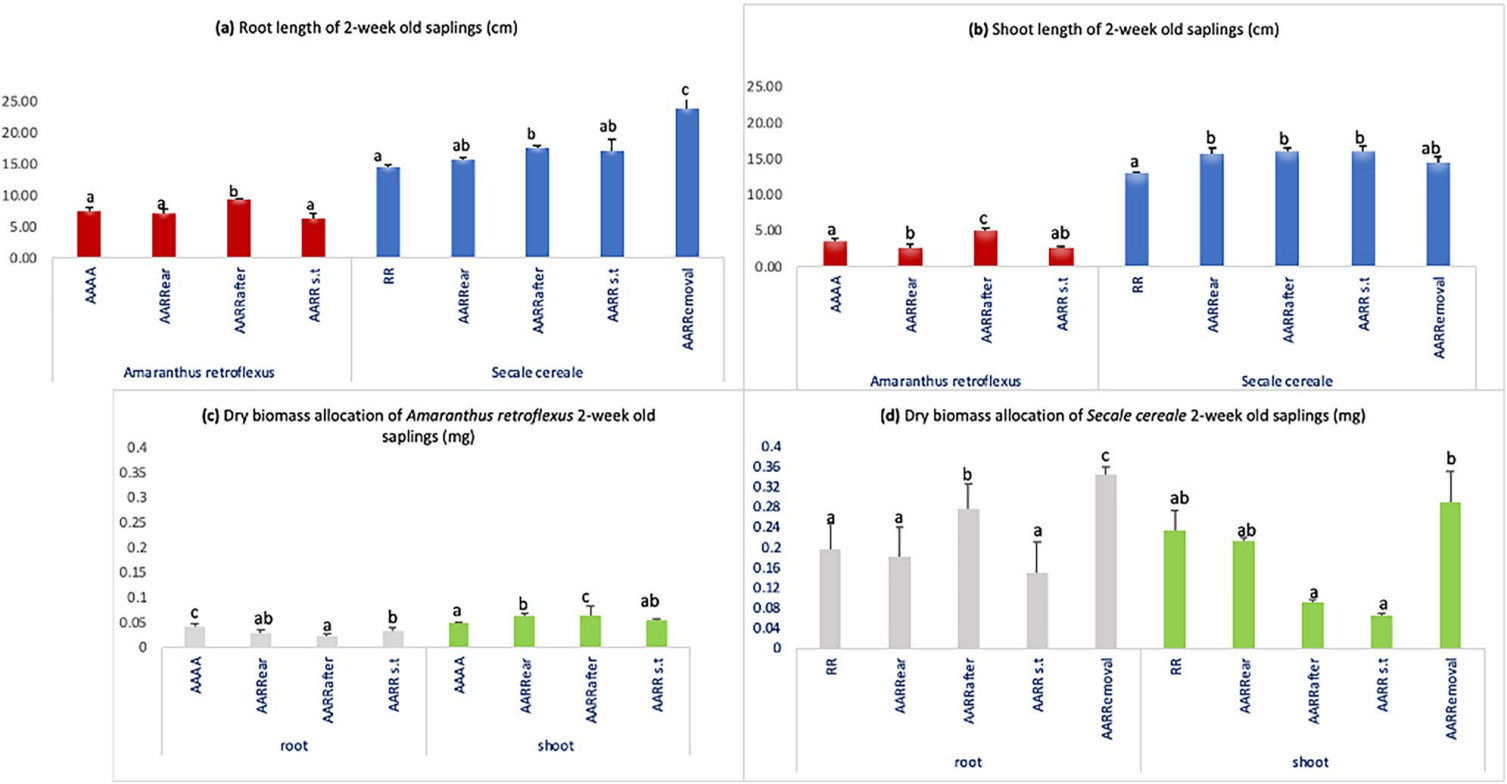
Growth comparison between 2 week-old pigweed and rye Plants: (**a**) Root and (**b**) Shoot Lengths (**c**) Dry biomass of pigweed plants (**d**) Dry biomass of rye plants. Values on the graphs represent means (average values) from five samples, with error bars showing standard errors (SE). Different letters above the columns indicate significant differences among different modalities, with significance at p < 0.05 according to the Tukey test.

After 2 weeks of growth, we did not see any difference in pigweed root length in any modality except for AARRafter which showed improved growth (Fig. 1a), and a reduction in its shoot length in AARRear when compared to control (AAAA) (Fig. 1b). Surprisingly, AARRafter shows improved growth (both root and shoot) (Fig. 1a,b). On the contrary, rye shows more or less improved seedling length (SL) in all modalities when compared with the control (RR) (Fig. 1a,b) with AARRemoval showing maximum root growth (Fig. 1a).

In terms of dry biomass, we observed a significant reduction in pigweed root in all modalities in co-culture with rye, in comparison to its control (AAAA). However, for shoots biomasses were significantly higher for AARRear and AARRfter (Fig. 1c) with AARRafter showing maximum growth which is in line with the observations in Fig. 1b,d shows the dry biomass allocation in rye wherein AARRemoval shows the highest root shoot biomass among all modalities.

### Seedling length and dry biomass allocation of 4 week-old plants

A reduction in pigweed root length is observed in all modalities except AARRafter in contrast to control (AAAA). Pigweed shoot length displays similar results. Almost, the same trend was observed at the 2 week-old stage wherein AARRafter showed the highest growth.

**Figure 2:**
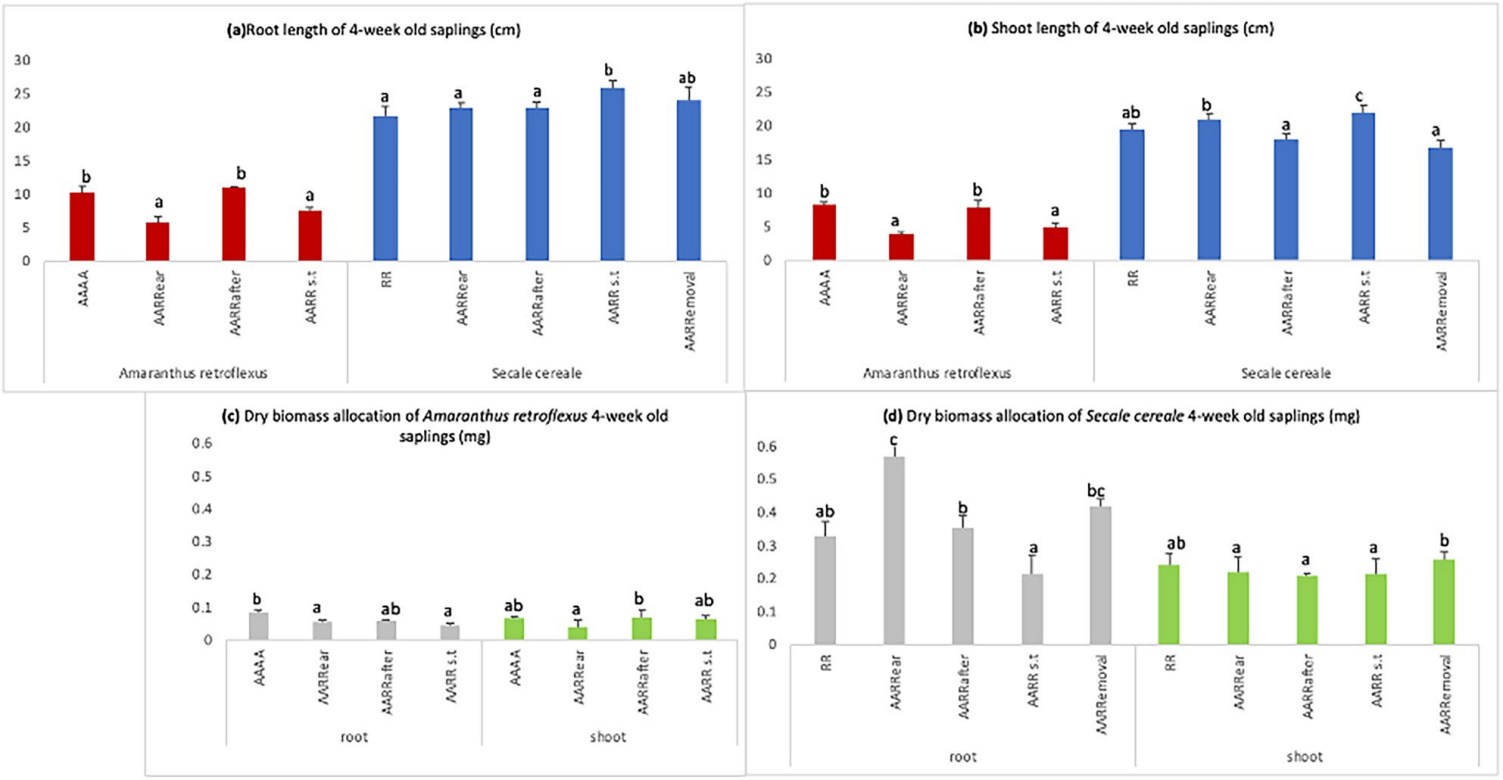
Growth comparison between 4 week-old pigweed and rye Plants: (**a**) Root and (**b**) Shoot Lengths (**c**) Dry biomass of pigweed plants (**d**) Dry biomass of rye plants. Values on the graphs represent means (average values) from five samples, with error bars showing standard errors (SE). Different letters above the columns indicate significant differences among different modalities, with significance at p < 0.05 according to the Tukey test.

Rye did not show any major changes in root length in all modalities except AARRs.t showing improved growth when compared with the control (RR) (Fig. 2a). Moreover, AARR s.t shows the maximum shoot length (Fig. 2b).

Reduction in dry biomass of pigweed root was observed in all modalities except in AARRafter (Fig. 2c), Moreover, the shoot shows improved biomass with AARRafter which coincides with our results of the 2 week-old experiment. Rye in AARRear shows the highest root biomass followed by AARRemoval in contrast to the control (Fig. 2d). Similarly, AARRemoval (and control) shows the highest shoot biomass which coincides with our results of the 2 week-old experiment.

### Germination experiments

**Figure 3:**
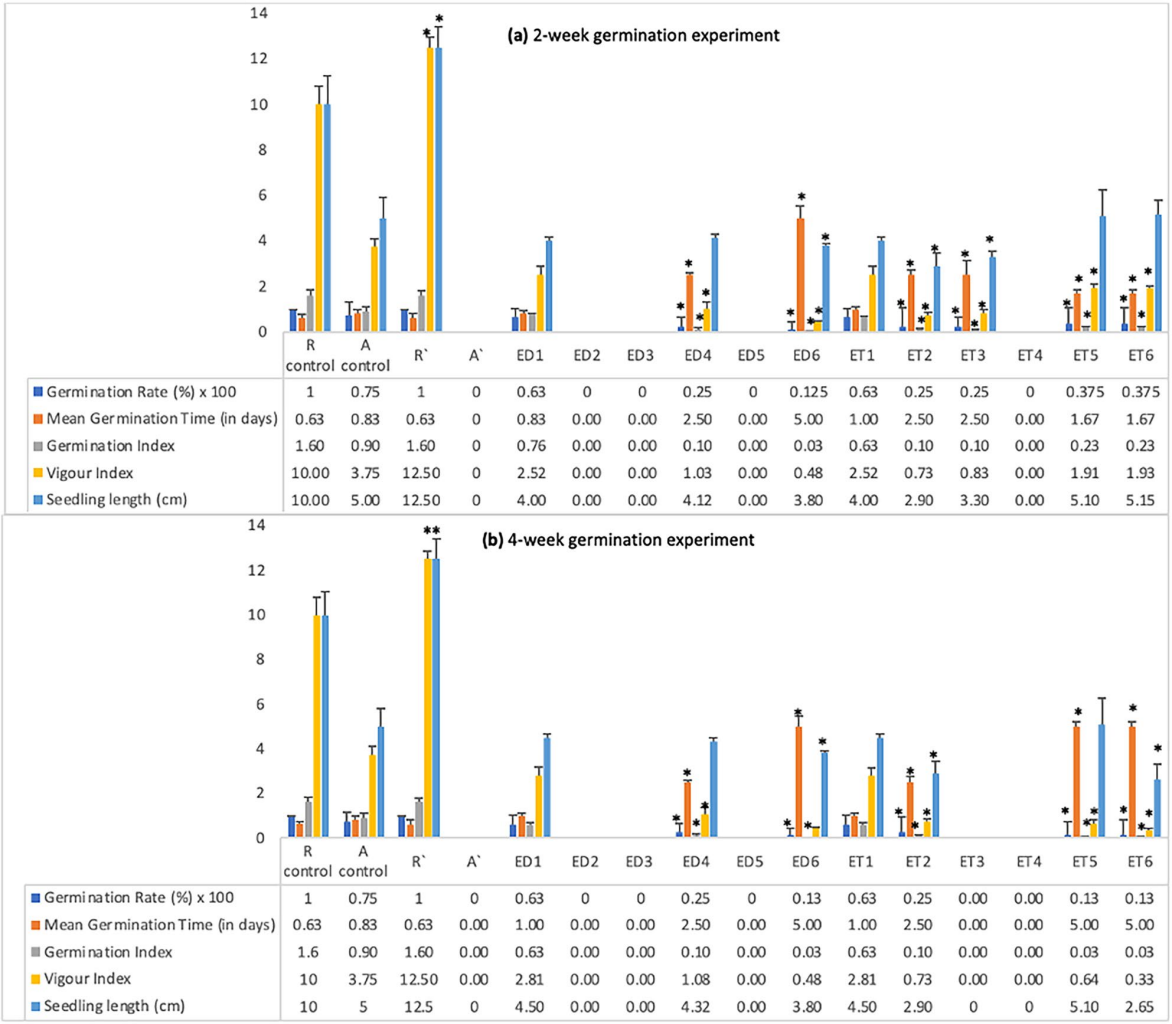
Germination indices of rye-pigweed co-germination and pigweed germination against (**a**) 2 week-old exudates and extracts (**b**) 4 week-old exudates and extracts. Values plotted are means (n = 5) ± standard errors (SE). R′ represents rye germination compared to its control ‘R control’ and A′ represents pigweed germination compared to its control ‘A control’ in the co-germination test. All other treatment groups ranging from ‘ED1 to ED6’, and ‘ET1 to ET6’ represent pigweed germination indices compared to their control ‘A control’. ‘*’ above columns represent significant differences in treatment groups from control at p < 0.05, applying Dunnett’s test.

In the co-germination test, rye shows a 100% germination rate (GR) both in control (R control) and in the presence of pigweed (represented by R′) (Fig. 3a,b). Moreover, rye shows improved vigour index (VI) and SL in the presence of pigweed. However, pigweed which showed 75% GR in control (A control) did not germinate in the presence of rye (A′).

On exposure to some 2 week-old exudates (ED2, ED3, ED5) and extracts (ET4) pigweed seeds did not germinate (Fig. 3a). Pigweed showed the highest GR in control (75%) followed by ED1 = ET1 (63%), ET5 = ET6 (38%), ED4 = ET2 = ET3 (25%) and ED6 (13%) (Fig. 3a). In general, mean germination time (MGT) tends to increase while germination index (GI) and vigour index (VI) decrease when pigweed is exposed to exudates and extracts when compared with control.

Figure 3b represents the germination indices of the 4 week germination experiment. Pigweed shows no germination when exposed to exudates (ED2, ED3, ED5) and extracts (ET3, ET4), very similar to the 2 week germination experiment. Pigweed shows the highest GR in control (75%) followed by ED1 = ET1 (63%), ED4 = ET2 (25%), and ED6 = ET5 = ET6 (13%). It appears that plant extracts from 4 week-old samples have stronger inhibitory potential than their 2 week-old counterparts. MGT, GI and VI follow a similar trend as observed with the 2 week germination experiment. ED1 and ET1 (corresponding to the modality AAAA) both at the 2 week and 4 week stages showed no significant difference statistically in pigweed germination indices when compared to the control. Moreover, the observed value of osmotic potential (OP) of plant extracts had no significant impact on pigweed germination (Table S1 and Fig. S3).

### HPLC-DAD analysis

In our study, we focused on the analysis of BXs (DIBOA, DIMBOA and MBOA) in the context of allelopathic interactions between rye and pigweed. No BXs were detected in the root exudates of both 2 week and 4 week-old samples. It may be because BXs were below our LOD (5.00 μg/g for DIBOA, 12.00 μg/g for DIMBOA and 6.05 μg/g for MBOA) or altogether not present.

**Figure 4:**
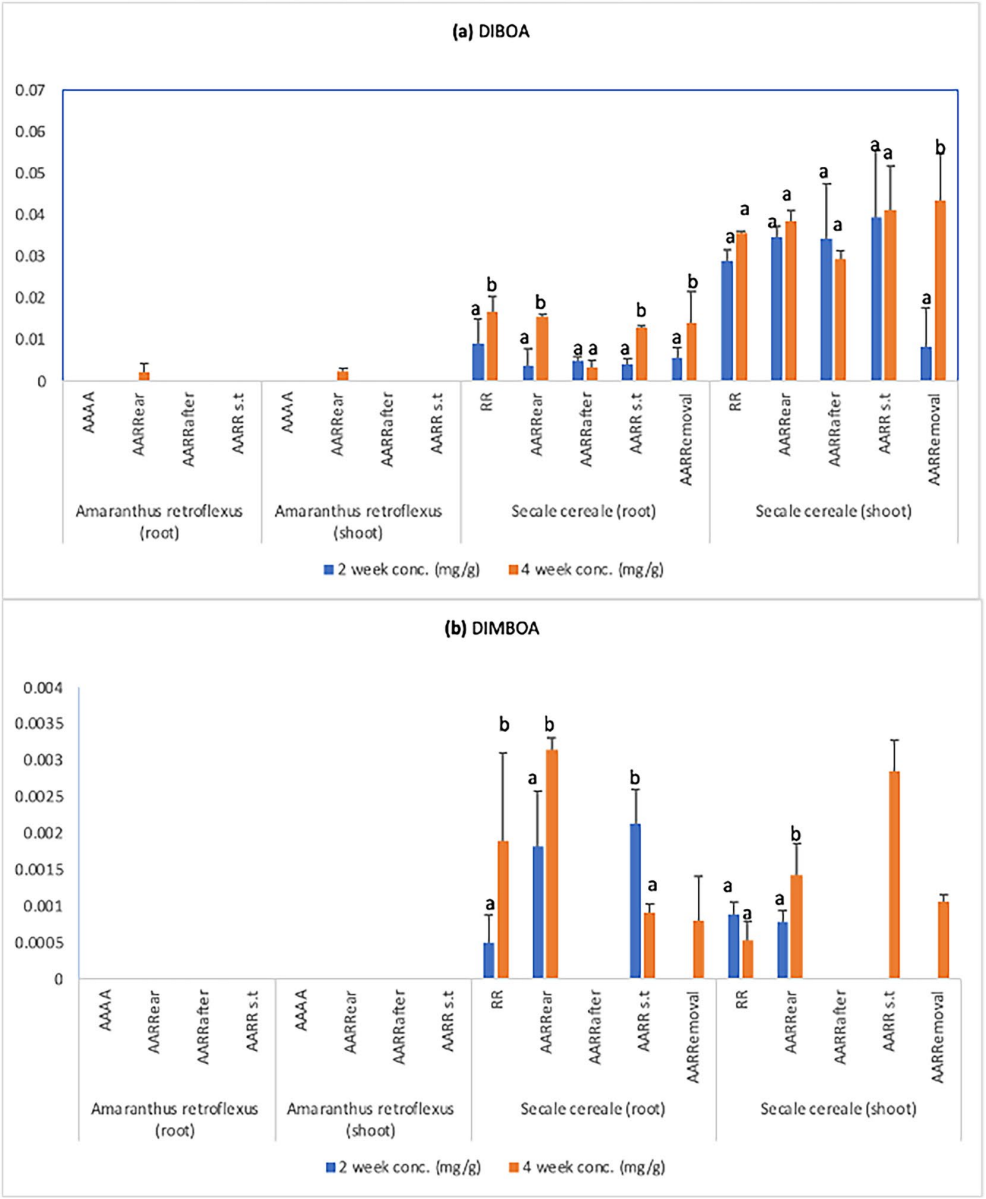
Concentration of BXs (**a**) DIBOA and (**b**) DIMBOA in the plant material. Values plotted are means (n = 5) ± standard errors (SE). Columns with different letters indicate significant differences among two and 5 week-old samples at p < 0.05 according to the Tukey test. Columns with no letters indicate the presence of DIMBOA only at the 4 week stage.

In the plant material, there were no BXs detected in the control group of pigweed (AAAA). Moreover, MBOA was not detected in any of our plant samples. DIBOA (shown in Fig. 4a) was found in all 2 week and 4 week-old rye plants. The concentration of DIBOA was higher in the shoots than in the roots. In addition, there was a statistically significant increase in the concentration of DIBOA in 4 week-old root samples compared to the 2 week-old samples. The modality “AARRemoval” had the highest concentration of DIBOA in the shoots at the 4 week-old stage.

The concentration of DIBOA in rye increases as the plant biomass increases (Fig. 2d). It’s interesting to note that rye seems to benefit from increasing DIBOA concentration. DIBOA was detected in some 4 week-old pigweed samples in the modality “AARRear”. This indicates that pigweed may have taken up BXs, suggesting a possible interaction between DIBOA and pigweed. We have mixed results with DIMBOA. Unlike DIBOA, DIMBOA (shown in Fig. 4b) was not found in any pigweed samples that were grown together with rye and was not found in all rye plant samples. However, like DIBOA, in some rye root samples, DIMBOA was more concentrated at the 4 week growth stage. In the modalities, AARRs.t and AARRemoval, DIMBOA were only detected in 4 week-old rye shoots.

## Discussion

Rye has been previously shown to produce and exude benzoxazinoids (BXs)^10–12^, however, the extent of their mediation in plant-plant interactions remains unclear. In the initial phase of our study (2 week stage), we observed intriguing patterns in pigweed and rye SL and biomass allocation. Despite no reduction in pigweed root length in any modality, shoot length decreased when rye was introduced earlier (AARRear) compared to the control (AAAA). Since the plants were continuously supplied by Hoagland solution, we can eliminate resource competition and consider chemical interference in our study, indicating allelopathic effects on pigweed SL aligning with previous research on rye’s impact on neighbouring plants^19,20^. Rye’s allelopathic impact on pigweed seems to primarily affect above-ground growth at this early stage. Rye is known for reducing the plant growth of its neighbours by releasing BXs^21^. This reduction is associated with BXs inhibiting mitotic activity and disrupting nucleus, mitochondria and chloroplast functions^22^. Interestingly, the AARRafter modality shows improved growth in pigweed, especially in shoot length, highlighting the nuanced influence of timing in seed sowing on crop-weed interactions. The influence of seed-sowing timing on crop-weed interactions is a well-explored area in agricultural research^23^. On the contrary, rye shows more or less improved SL in all modalities when compared with the control (RR) (Fig. 1a,b) especially in the AARRemoval modality (Fig. 1a), indicating a potential case of induced allelopathy in rye after a brief introduction of pigweed (further supported by increased BXs concentration in rye exposed to pigweed, discussed later). As the experiment progressed to the 4 week stage, the trends observed in SL and biomass allocation continued to offer insights into the dynamics of the rye-pigweed interaction. Pigweed root length reduction persisted across modalities, except for AARRafter (Fig. 2a), while shoot length variations mirrored those of the 2 week stage, with AARRafter displaying the highest growth (Fig. 2b). This consistency in results across different growth stages highlights the robustness of the allelopathic effects of rye on pigweed and the importance of differential seed-sowing time^17^. Rye, once again, displayed no significant changes in root length across modalities, with AARRs.t showed improved growth compared to the control. The significant growth of pigweed observed in the AARRafter configuration is intriguing and deserves further investigation to understand the underlying mechanisms.

The biomass allocation results add depth to the understanding of the allelopathic interactions^24^. The reduction in pigweed’s root biomass in all modalities at the 2 week stage (Fig. 1c) indicates that rye’s presence might primarily affect the root system of pigweed^25^. This could be due to thinning of pigweed roots (which explains no significant reduction in root lengths). This contrasts with the increased root biomass observed in the rye, particularly in AARRafter and AARRemoval (Fig. 1d), further suggesting the concept of induced allelopathy^23^. At the 4 week stage, the biomass allocation results mirrored those of the 2 week stage, with pigweed’s root biomass reduced in all modalities except AARRafter, while shoot biomass did not show any decline when compared to control, aligning with the 2 week results (Fig. 2c). Our findings related to rye’s biomass allocation in AARRear and AARRemoval are noteworthy (Fig. 2d). These modalities exhibit increased root and shoot biomass, indicating a complex interplay of allelopathic effects. The variation in the presence of pigweed in the growth medium proves to be another significant factor that has influenced the interactions and outcomes of our co-culture^26^.

In the germination experiments, rye exhibited 100% germination regardless of the presence of pigweed. This suggests that rye’s germination is not affected by the presence of pigweed. While there are studies showing pigweed plant extracts inhibiting crop-germination^27,28^, however, there is no report of crop-germination inhibition in crop-pigweed co-culture. In contrast, pigweed, with a 75% GR in control, failed to germinate in the presence of rye. Exposure of pigweed seeds to 2 week-old exudates and extracts revealed inhibitory effects on germination, with varying germination rates across treatments. The 4 week germination experiment reinforced these findings, showing stronger inhibitory potential in plant exudates and extracts collected from 4 week-old samples compared to their 2 week counterparts. This aligns with previous studies demonstrating rye’s potent inhibitory effect on pigweed germination^29,30^. Another study shows inhibition of radish seed germination due to the absorption of BXs released by rye^31^. This consistency in results suggests that rye can detect its neighbours as early as at the germination stage and releases certain substances in response that inhibit their germination^29–31^. Several studies have reported seed germination inhibition due to BXs inhibiting α-amylase activity^7,32,33^. The absence of significant differences in pigweed germination indices between ED1 = ET1 and the control suggests that the observed germination inhibition is due to rye BXs and not pigweed autotoxicity. Moreover, in our study, the MGT tended to increase while GI and VI decreased when pigweed was exposed to exudates and extracts compared to control conditions. This trend indicates a delay in germination and reduced seedling vigour when pigweed interacts with rye exudates/extracts.

HPLC-DAD analysis provides insights into chemicals (BXs) potentially responsible for the above-discussed reduction in pigweed growth and germination. No BXs were found in the root exudates of either 2 week or 4 week-old samples. However, the results from the germination experiments establish their allelopathic potential. This suggests that root exudates may have BXs below the LOD and our system could not detect them. DIBOA was detected in all rye plants, with a significant increase in co-cultivation with pigweed, supporting the concept of induced allelopathy. Moreover, this increase in DIBOA concentration aligns with the observed positive effects on rye growth. Our observation is consistent with the results of Hazrati et al.^12^ who showed increased BX production in rye in response to cocultivation with *Vicia villosa* L. Furthermore, the concentration of DIBOA is higher in the rye shoots than in the roots, suggesting more synthesis of this compound in shoots or its vigorous transport from root to shoot. This finding is supported by the results of Rice et al.^34^ which shows DIBOA to be a shoot-dominant compound in rye. The “AARRemoval” modality had the highest concentration of DIBOA in the rye shoots at the 4 week-old stage, indicating that removing pigweed after its brief exposure to rye may trigger increased production of allelopathic compounds, emphasizing the dynamic nature of these interactions. The age-dependent dynamics of allelopathic compounds in rye were evident in the increase in DIBOA concentration from 2 week to 4 week samples^34^. The presence of DIBOA in some 4 week-old pigweed samples in the modality “AARRear” suggests pigweed may take up BXs produced by rye^12^, indicating a potential interaction between DIBOA and pigweed. DIMBOA was not found in all rye samples, suggesting it may not play a significant role in allelopathic interactions in this co-culture.

Conclusively, the juxtaposition of growth patterns and biomass allocation at the 2 week and 4 week stages in the rye-pigweed co-culture, along with the scrutiny of BXs concentration and germination evaluation, emphasizes the enduring allelopathic impact of rye on pigweed. The identified trends in growth patterns and biomass allocation yield a comprehensive grasp of the dynamic nature of these interactions, suggesting potential enhancements in crop-weed dynamics within agroecosystems. The association with BX concentration reinforces the concept of induced allelopathy, opening avenues for prospective investigations into the mechanisms governing the production and absorption of allelopathic compounds. The AARRafter and AARRemoval configurations emerge as promising avenues for deeper exploration into the underlying mechanisms. These revelations enrich our comprehension of allelopathic dynamics in agroecosystems, carrying practical implications for weed control strategies and crop enhancement.

## Methods

### Experimental design

Pigweed (A) and rye (R) seeds were treated with a 0.2% sodium hypochlorite solution for 10 min and washed three times with distilled water to prevent contaminations^34^. Then, both types of seeds were allowed to germinate and grow in different arrangements and combinations (Fig. S1): (i) pigweed alone (4 seeds) (AAAA), (ii) rye alone (2 seeds) (RR), (iii) rye (2 seeds) and pigweed (2 seeds) sown at the same time (AARR s.t), (iv) rye (2 seeds) sown 3 days earlier than pigweed (2 seeds) (AARRear), (v) rye (2 seeds) sown 3 days after pigweed (2 seeds) (AARRafter), and (vi) pigweed (2 seeds) sown first, removed after one week, and then rye (2 seeds) sown (AARRemoval). Seeds were sown in plastic tubes (Empty SPE 60 mL cartridge with two pre-inserted 20 µm frits by Agilent^®^) covered with black tape for opacity. The tubes were filled with sandglass (BlasterGlass (granulometry 250–425 µm, no free silica)) and were moisturized with 5 mL of half-strength Hoagland solution every alternate day until 2 days before analysis (to allow the drying of the medium and thus avoid as much as possible the extraction of nutritive residues). The entire set-up was maintained in a Phytotron conditioned to 12 ho day/12 h night photoperiod, 21 °C day/18 °C night thermoperiod and 70% relative humidity. Analyses were conducted after 2 and 4 weeks of growth. Monitoring at 2 and 4 weeks allows us to capture dynamic changes (SL dry biomass, BX production) over time in an age-dependent manner.

Fifteen replicates were maintained for each modality. Five of them were used for root exudate collection (as described in “Collection of root exudates” section) followed by SL, and dry biomass analysis (“Seedling length and dry biomass” section) (Fig. S1). Five replicates were used for aqueous extract collection (“Preparation of aqueous extracts” section). The collected root exudates and aqueous extracts were also used in the subsequent germination tests (“Germination test” section). The remaining 5 replicates were used for quantification of benzoxazinoids (BXs) by high-performance liquid chromatography coupled to UV absorption detector and diode array detector (HPLC-UVD) analysis (“Sample preparation for HPLC-UVD analysis”, Quantification of benzoxazinoids in plant material and root exudate by HPLC-UVD analysis” sections).

#### Collection of root exudates

Root exudates were collected according to the protocol of Hazrati et al.^12^ using a customised device as shown in Fig. S2. This method was adopted to mimic the leaching of allelochemicals from root surfaces under natural conditions. A solvent containing 70% methanol (v/v) (HPLC grade > 99%) (Honeywell, France) and 0.2% formic acid (v/v) (Sigma Aldrich, Germany) was used for root exudate extraction. The choice of the solvent at 70% is to avoid the extraction of intracellular compounds^35^ and therefore does not enter the frame of this work. Fifteen millilitres of extraction solution were injected manually with a syringe into the top of the tube in 30 s with a flow rate of 1 mL/s in a way to avoid contact with the stem of the plants. Each extraction was carried out under a vacuum of 780 mbar held for one minute to accelerate the flushing of the solution. The freshly collected root exudates were filtered through a 0.22 μm cellulose acetate syringe filter. A part of the collected exudates was transferred into glass vials before HPLC analysis and the other half was kept for germination tests.

#### Seedling length and dry biomass

After exudate collection, the seedlings were carefully taken out of the plastic tubes and soaked in stirred deionized water for approximately one minute. This step allows the glass beads to detach from the roots and helps the roots of plants in co-culture to separate from each other as well. Thereafter, the growth parameters (root/shoot length) of each plant were measured using a meter scale before cutting it using scissors just above the root collar to segregate root and shoot systems. Collected samples were then dried in an electrical oven at 60 °C for 48 h and their dry mass was determined by weighing.

#### Preparation of aqueous extracts

The seedlings were carefully taken out of the plastic tubes and soaked in stirred deionized water for approximately one minute. The entire plant, along with roots and shoots from all co-cultured plants, was subjected to freeze-drying using liquid nitrogen, followed by lyophilization for 48 h. 4% aqueous extracts of each modality were prepared following the methodology described by Mushtaq et al.^36^. In modalities where two different plants (rye and pigweed) were growing together, an equal amount of both plant types in a 1:1 weight ratio was used to make aqueous extracts. This method was adopted to mimic the release of allelochemicals from entire plant tissue (including intracellular compounds) after plants die and decompose under natural conditions.

Moreover, the OP of extracts was determined using the formula: OP = 0.36 × Conductivity (mS) (Table S1). The conductivity was measured using a digital conductivity meter (Consort K610, Belgium) by immersing its electrode into each extract.

#### Germination test

Two types of germination tests were performed:

##### Co-germination of rye and pigweed

Rye and pigweed (5 seeds each) were allowed to germinate in the same Petri dish and in separate Petri dishes (to serve as control) lined with filter paper.

##### Allelopathic impact of root exudates and extracts on pigweed germination

Pigweed (5 seeds) were placed in Petri dishes lined with filter paper sprinkled with 5 mL of different exudates and extracts collected earlier (“Collection of root exudates” and “Preparation of aqueous extracts” sections) at 2 week and 4 week stages given in Table 1. This is to establish if any differences in the allelopathic potential between exudates and extracts collected at different growth stages.

**Table 1:** List of root exudates and aqueous extracts corresponding to their modalities used in germination experiments.

Modality	Root exudate name	Aqueous extract name
AAAA	ED1	ET1
RR	ED2	ET2
AARR s.t	ED3	ET3
AARRear	ED4	ET4
AARRafter	ED5	ET5
AARRemoval	ED6	ET6

All the Petri dishes from both types of germination experiments (“Co-germination of rye and pigweed” and “Allelopathic impact of root exudates and extracts on pigweed germination” sections) were sprinkled with 10 ml of half-strength Hoagland solution, wrapped with black tape and maintained in an incubator at 25  ±  0.5 °C and 85% humidity. For each treatment, five replicates were made and the whole setup was retained in a completely randomized block design (CRBD). Two millilitres of Hoagland solution were added every 48 h to maintain the humidity of the filter paper in the Petri dish. The number of the germinated seeds was counted from the second day after treatment and the count lasted for one week. In addition, the root length, stem length and biomass of each germinated seedling were also measured.

The GR, MGT, GI and VI were calculated using the following equations^37^:

1 $$\left( \% \right) = Ni/N \times {1}00$$

2 $$MGT = \sum (d \times n)/\sum n$$

3 $$GI = \sum (d/n)$$

4 $$VI = GR \times (LR + LP)$$

where *Ni* is the number of germinated seeds on the 7th day, *N* is the total seed number in the petri dish, *Nt* is the number of germinated seeds when the daily germination number reaches the peak, *d* is the number of seeds emerging on a given day, *n* is the time after setting the seeds for germination. Moreover, SL is also measured at the end of the 7th day as the sum of radicle length and plumule length.

Similarly, in a parallel experiment, we prepared mannitol solutions with corresponding OPs to that of plant extracts (from Table S1) and assessed their impact on pigweed germination against distilled water as a control (Fig. S3). Five replicates were maintained for each treatment. Mannitol was used in this study because it has been previously demonstrated to act as an inert osmotic medium for such studies^38^.

#### Sample preparation for HPLC-UVD analysis

HPLC analyses were performed on root exudates collected as described in “Collection of root exudates” section but also directly with plant material. In this second case, after having carefully taken out the seedlings of the plastic tubes, they are soaked and stirred in deionized water for approximately one minute to separate the roots of plants in co-culture and get rid of attached glass beads. In each modality, the root was separated from the stem of all seedlings as explained in “Seedling length and dry biomass” section. The plant material was immediately freeze-dried using liquid nitrogen followed by lyophilization for 48 h before sample preparation for analysis.

A weight of 25 mg of the plant material was crushed and placed in Eppendorf tubes. To this, 1 mL of extraction solvent (a mix of methanol, water, and formic acid in the ratio 50:50:1, v/v/v) was added along with four glass beads (each smaller than 1 mm). The mixture was agitated for 1 h using a Heindolph Multireax Agitator set at 2000 rpm. After agitation, the solid phase was separated using an Eppendorf MiniSpin centrifuge at 13,400 rpm for 8 min The supernatant was then drawn with a syringe, filtered through a 0.45 μm PTFE filter into a vial, and stored at 4 °C before analysis. The root exudates collected in “Collection of root exudates” section shall be used as such for the analysis.

#### Quantification of benzoxazinoids in plant material and root exudate by HPLC-UVD analysis

The benzoxazinoids quantification was done on an Agilent 1200 HPLC System. Separation was done on an Agilent Poroshell C18 column and using Solution A (methanol/water/ortho-phosphoric acid 85%; 10/90/0.1; v/v/v) and Solution B (methanol/ortho-phosphoric acid 85%; 100/0.1; v/v) as eluents. The injection volume was 10 μL. Quaternary pump programming is given in Table S2. Before each run of samples, a set of standard solutions was injected to confirm retention times. The standards used are DIBOA, DIMBOA and MBOA (6-methoxy-benzoxazolin-2-one) purchased from Sigma-Aldrich. Absorption was measured at 250 nm; 280 nm and 288 nm for DIBOA, DIMBOA and MBOA respectively. The standard deviation (S) of the measured concentrations was calculated for each compound, the limit of detection (LOD) was determined as 3 × S, and the limit of quantification (LOQ) as 10 × S^12^. Quantifications of BXs in plant material and root exudates were done based on the standard curves prepared for our standard compounds. Data points of the standard curves were weighted according to x^–1^.

### Statistical analysis

All data analysis was performed with RStudio software (Version 2023.09.0+463). To compare the SL, dry biomass and concentration of BXs between treatments, a T-test and one-way analysis of variance (ANOVA) were applied followed by the Tukey honestly significant difference (HSD) test at a significance level of 95% to compare each group with the other groups. The data were checked for a normal distribution. For germination parameters, the data was fitted into a binomial GLM (generalized linear model) with a logit link function (Fig. 3), and Dunnett’s test was applied at a significance level of 95%.

### Ethical approval

We had permission to buy/collect seeds used in this study. All the methods used in this study were carried out in accordance with relevant guidelines and regulations.

### Supplementary Information

Supplementary Information.

## Data Availability

All data generated or analysed during this study are included in this published article (and its Supplementary Information files).

## References

[1] San Emeterio L, Damgaard C, Canals RM (2007). Modelling the combined effect of chemical interference and resource competition on the individual growth of two herbaceous populations. Plant Soil..

[2] Gfeller A, Glauser G, Etter C, Signarbieux C, Wirth J (2018). *Fagopyrum esculentum* alters its root exudation after *Amaranthus retroflexus* recognition and suppresses weed growth. Front. Plant Sci..

[3] Uesugi A, Johnson R, Kessler A (2019). Context-dependent induction of allelopathy in plants under competition. Oikos..

[4] Delory BM, Delaplace P, Fauconnier ML, Du Jardin P (2016). Root-emitted volatile organic compounds: Can they mediate belowground plant-plant interactions?. Plant Soil.

[5] Kong CH, Zhang SZ, Li YH, Xia ZC, Yang XF, Meiners SJ, Wang P (2018). Plant neighbour detection and allelochemical response are driven by root-secreted signalling chemicals. Nat. Commun..

[6] Hussain MI, Araniti F, Schulz M, Baerson S, Vieites-Álvarez Y, Rempelos L, Sánchez-Moreiras AM (2022). Benzoxazinoids in wheat allelopathy–from discovery to application for sustainable weed management. Environ. Exp. Bot..

[7] Hu L, Robert CA, Cadot S, Zhang XI, Ye M, Li B, Erb M (2018). Root exudate metabolites drive plant-soil feedbacks on growth and defense by shaping the rhizosphere microbiota. Nat. Commun..

[8] Belz RG, Hurle K (2005). Differential exudation of two benzoxazinoids: One of the determining factors for seedling allelopathy of *Triticeae* species. J. Agric. Food Chem..

[9] Pratt K, Kumar P, Chilton WS (1995). Cyclic hydroxamic acids in dicotyledonous plants. Biochem. Syst. Ecol..

[10] Rakoczy-Trojanowska M, Szabała BM, Różańska E, Kowalczyk M, Burza W, Bakera B, Swięcicka M (2021). The roots of rye (*Secale cereale* L.) are capable of synthesizing benzoxazinoids. Int. J. Mol. Sci..

[11] Rice CP, Otte BA, Kramer M, Schomberg HH, Mirsky SB, Tully KL (2022). Benzoxazinoids in roots and shoots of cereal rye (*Secale cereale*) and their fates in soil after cover crop termination. Chemoecology.

[12] Hazrati H, Fomsgaard IS, Kudsk P (2020). Root-exuded benzoxazinoids: Uptake and translocation in neighbouring plants. J. Agric. Food Chem..

[13] Li A, Zheng R, Tian L, Wei Y, Wu J, Hou X (2021). Allelopathic effects of switchgrass on redroot pigweed and crabgrass growth. Plant Ecol..

[14] Knez̆ević SZ, Weise SF, Swanton CJ (1995). Comparison of empirical models depicting density of *Amaranthus*
*retroflexus* L and relative leaf area as predictors of yield loss in maize (Zea mays L.). Weed Res..

[15] Bakhshayeshan-Agdam H, Salehi-Lisar SY, Motafakkerazad R, Talebpour A, Farsad N (2015). Allelopathic effects of redroot pigweed (*Amaranthus*
*retroflexus* L.) on germination & growth of cucumber, alfalfa, common bean and bread wheat. Acta. Agric. Slov..

[16] Mirshekari B (2011). Effects of density and date of emergence of redroot pigweed (*Amaranthus*
*retroflexus* L.) on sugar beet (*Beta*
*vulgaris* L.) yield. Agroecol. J..

[17] Amini R, Alizadeh H, Yousefi A (2014). Interference between red kidneybean (*Phaseolus*
*vulgaris* L.) cultivars and redroot pigweed (*Amaranthus*
*retroflexus* L.). Eur. J. Agron..

[18] Knezevic SZ, Weise SF, Swanton CJ (1994). Interference of redroot pigweed (*Amaranthus retroflexus*) in corn (Zea mays). Weed Sci..

[19] Adhikari L, Mohseni-Moghadam M, Missaoui A (2018). Allelopathic effects of cereal rye on weed suppression and forage yield in *Alfalfa*. Am. J. Plant Sci..

[20] Tabaglio V, Gavazzi C, Schulz M, Marocco A (2008). Alternative weed control using the allelopathic effect of natural benzoxazinoids from rye mulch. Agron. Sustain. Dev..

[21] Zhou S, Richter A, Jander G (2018). Beyond defense: Multiple functions of benzoxazinoids in maize metabolism. Plant Cell Physiol..

[22] Gniazdowska A, Bogatek R (2005). Allelopathic interactions between plants. Multi site action of allelochemicals. Acta. Physiol. Plant.

[23] Mir MS, Singh P, Bhat TA, Kanth RH, Nazir A, Al-Ashkar I, El Sabagh A (2023). Influence of sowing time and weed management practices on the performance and weed dynamics of direct drum seeded rice. ACS Omega.

[24] Rutherford MC, Powrie LW (1993). Allelochemic control of biomass allocation in interacting shrub species. J. Chem. Ecol..

[25] Hilhorst HW, Toorop PE (1997). Review on dormancy, germinability and germination in crop and weed seeds. Adv. Agron..

[26] Hassan MM, Daffalla HM, Yagoub SO, Osman MG, Gani MEA, Babiker AGE (2012). Allelopathic effects of some botanical extracts on germination and seedling growth of *Sorghum bicolor* L. J. Agric. Technol..

[27] Konstantinović B, Blagojević M, Konstantinović B, Samardžić N (2014). Allelopathic effect of weed species *Amaranthus retroflexus* L. on maize seed germination. Rom. Agric. Res..

[28] Mlakar SG, Jakop M, Bavec M, Bavec F (2012). Allelopathic effects of *Amaranthus retroflexus* and *Amaranthus cruentus* extracts on germination of garden cress. Afr. J. Agric. Res..

[29] La Hovary C, Danehower DA, Ma G, Reberg-Horton C, Williamson JD, Baerson SR, Burton JD (2016). 2016 Phytotoxicity and benzoxazinone concentration in field grown cereal rye (*Secale cereale* L.). Int. J. Agron.

[30] Flood HE, Entz MH (2009). Effects of wheat, triticale and rye plant extracts on germination of navy bean (*Phaseolus vulgaris*) and selected weed species. Can. J. Plant Sci..

[31] Chiapusio G, Pellissier F, Gallet C (2004). Uptake and translocation of phytochemical 2-benzoxazolinone (BOA) in radish seeds and seedlings. J. Exp. Bot..

[32] Kato-Noguchi H, Macias FA (2008). Inhibition of germination and α-amylase induction by 6-methoxy-2-benzoxazolinone in twelve plant species. Biologia Plantarum.

[33] Ozaki Y, Kato-Noguchi H (2016). Effects of benzoxazinoids in wheat residues may inhibit the germination, growth and gibberellin-induced α-amylase activity in rice. Acta Physiol. Plant..

[34] Li J, Chen L, Chen Q, Miao Y, Peng Z, Huang B, Du H (2021). Allelopathic effect of *Artemisia*
*argyi* on the germination and growth of various weeds. Sci. Rep..

[35] Petriacq P, Williams A, Cotton A, McFarlane AE, Rolfe SA, Ton J (2017). Metabolite profiling of non-sterile rhizosphere soil. Plant J..

[36] Mushtaq W, Ain Q, Siddiqui MB, Hakeem KR (2019). Cytotoxic allelochemicals induce ultrastructural modifications in *Cassia tora* L. and mitotic changes in *Allium cepa* L.: A weed versus weed allelopathy approach. Protoplasma.

[37] Zhao J, Yang Z, Zou J, Li Q (2022). Allelopathic effects of sesame extracts on seed germination of moso bamboo and identification of potential allelochemicals. Sci. Rep..

[38] Bell DT (1974). The influence of osmotic pressure in tests for allelopathy. 19750732479 Engl. J. Artic. Trans. Ill. State Acad. Sci..

