# Supplementary material for: Assessment of induced allelopathy in crop-weed co-culture with rye-pigweed model
Source: Sci Rep. 2024 May 7;14:10446. doi: 10.1038/s41598-024-60663-w (PMC11076540; doi:10.1038/s41598-024-60663-w)
Supplement: Supplementary file 1 — Supplementary Information. [file 41598_2024_60663_MOESM1_ESM.docx]

**Title: Assessment of induced allelopathy in crop-weed co-culture with rye-pigweed model**

Waseem Mushtaq*^1^, Marie-Laure Fauconnier**^1^, Caroline de Clerck**^2^

^1^Laboratory of Chemistry of Natural Molecules, Gembloux Agro-Bio Tech, Liege University, Passage des déportés 2, 5030 Gembloux, Belgium

^2^AgricultureIsLife, Gembloux Agro-Bio Tech, Liege University, Passage des déportés 2, 5030 Gembloux, Belgium

Coressponding author*: Waseem.Mushtaq@uliege.be

**Supplementary Tables:**

Table S1: Osmotic potential of plant extracts.

| Sampling time | Plant extracts | Osmotic potential (bars) |
| --- | --- | --- |
| 2-week | ET1 | 0.35 |
|  | ET2 | 0.48 |
|  | ET3 | 0.65 |
|  | ET4 | 0.55 |
|  | ET5 | 0.69 |
|  | ET6 | 0.5 |
| 4-week | ET1 | 0.4 |
|  | ET2 | 0.68 |
|  | ET3 | 0.68 |
|  | ET4 | 0.59 |
|  | ET5 | 0.7 |
|  | ET6 | 0.75 |

Table S2: Programmed sequence of the quaternary pumping system of Agilent 1200 HPLC system. Solution A (methanol/water/ortho-phosphoric acid 85%; 10/90/0,1; v/v/v) and Solution B (methanol/ ortho-phosphoric acid 85%; 100/0,1; v/v). The values represent the proportion of eluents pumped inside an Agilent Poroshell C18 separation column throughout the method.

| Time (min) | Solution A (%) | Solution B (%) |
| --- | --- | --- |
| 0.0 | 77.0 | 23.0 |
| 2.0 | 77.0 | 23.0 |
| 5.0 | 70.0 | 30.0 |
| 8.0 | 20.0 | 80.0 |
| 20.0 | 10.0 | 90.0 |
| 21.1 | 0.0 | 100.0 |
| 28.0 | 0.0 | 100.0 |
| 29.0 | 77.0 | 23.0 |
| 35.0 | 77.0 | 23.0 |

**Supplementary Figures:**


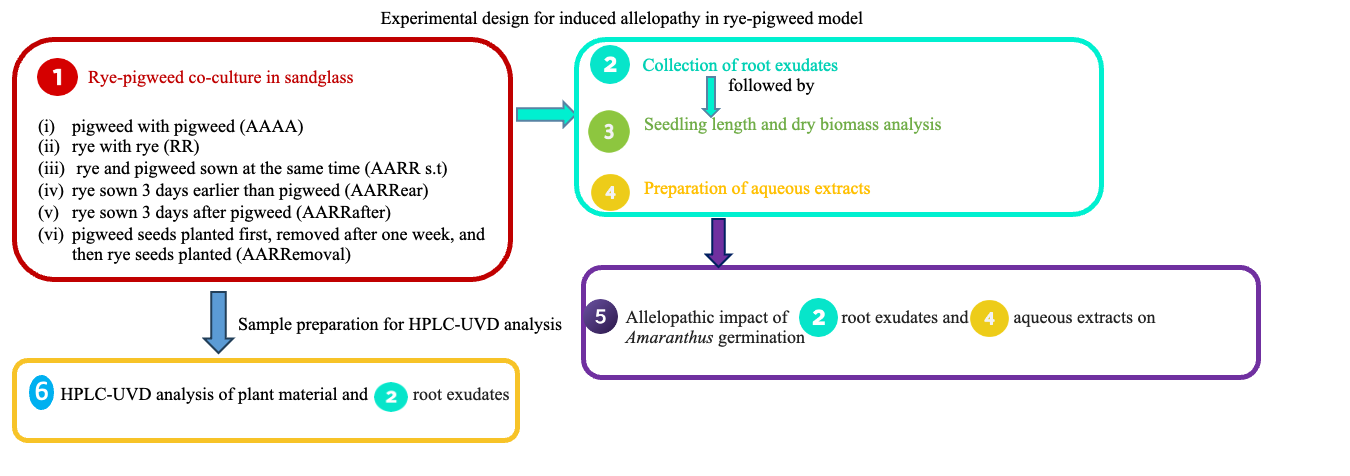


Fig. S1. Experimental design for rye-pigweed co-culture to assess induced allelopathy.


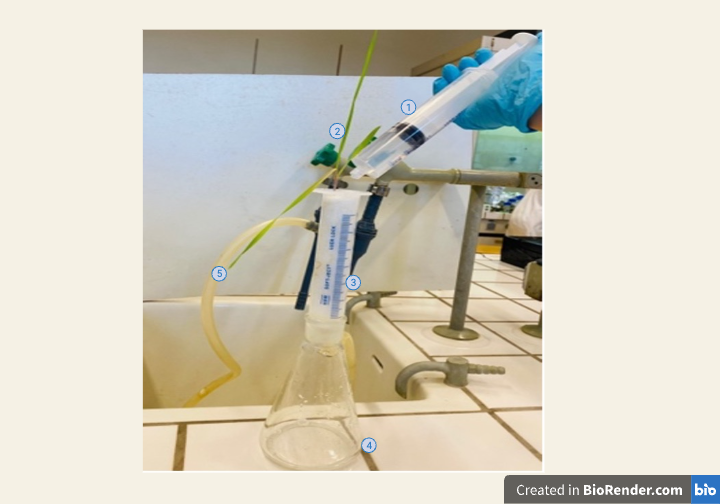


*Fig. S2. Customised device for root exudate extraction. (1) Extraction solvent is injected into the growing medium at the top in 30 seconds (2) Plant growing inside the medium (3) Growth medium (4) Root exudate collected (5) pipe connected to vacuum pump for maintaining the required pressure*


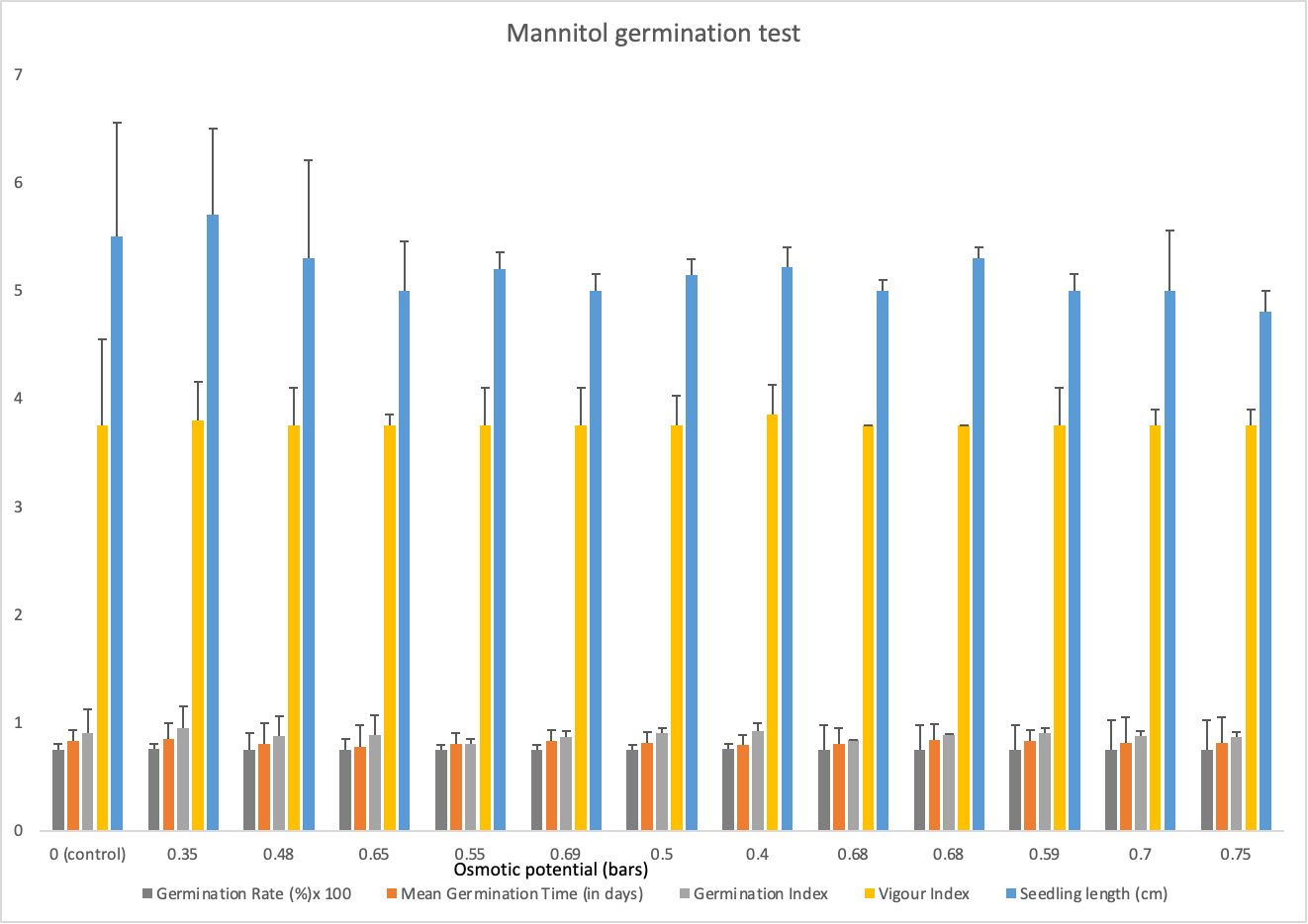


Fig. S3. Pigweed germination against mannitol solutions of osmotic potentials corresponding to plant extracts. Values plotted are means (n = 5) ± standard errors (SE). There was no significant difference between the means of different groups.
